# Urine NMR Metabolomics Profile of Preterm Infants With Necrotizing Enterocolitis Over the First Two Months of Life: A Pilot Longitudinal Case-Control Study

**DOI:** 10.3389/fmolb.2021.680159

**Published:** 2021-06-15

**Authors:** Jean-Charles Picaud, Anna De Magistris, Michele Mussap, Sara Corbu, Angelica Dessì, Antonio Noto, Vassilios Fanos, Flaminia Cesare Marincola

**Affiliations:** ^1^ Neonatology Unit, Croix-Rousse University Hospital, Hospices Civils de Lyon, Lyon, France; ^2^ Pediatrics and Neonatology Division of, Azienda USL Romagna, Santa Maria Delle Croci Hospital, Ravenna, Italy; ^3^ Department of Surgical Sciences, University of Cagliari, Cagliari, Italy; ^4^ Department of Biomedical Sciences, University of Cagliari, Cagliari, Italy; ^5^ Department of Chemical and Geological Sciences, Cittadella Universitaria di Monserrato, University of Cagliari, Cagliari, Italy

**Keywords:** metabolomics, proton nuclear magnetic resonance spectroscopy, necrotizing enterocolitis, prematurity, urine

## Abstract

**Objective:** To investigate changes in the urine metabolome of very low birth weight preterm newborns with necrotizing enterocolitis (NEC) and feed intolerance, we conducted a longitudinal study over the first 2 months of life. The metabolome of NEC newborns was compared with two control groups that did not develop NEC: the first one included preterm babies with feed intolerance, while the second one preterm babies with good feed tolerance.

**Methods:** Newborns developing NEC within the 3 weeks of life were identified as early onset NEC, while the remaining as late onset NEC. Case-control matching was done according to the gestational age (±1 week), birth weight (± 200 g), and postnatal age. A total of 96 urine samples were collected and analyzed. In newborns with NEC, samples were collected before, during and after the diagnosis over the first 2 months of life, while in controls samples were collected as close as possible to the postnatal age of newborns with NEC. Proton nuclear magnetic resonance (^1^H NMR) spectroscopy was used for metabolomic analysis. Data were analyzed by univariate and multivariate statistical analysis.

**Results:** In all the preterm newborns, urine levels of betaine, glycine, succinate, and citrate positively correlated with postnatal age. Suberate and lactate correlated with postnatal age in preterms with NEC and in controls with food intolerance, while *N,N*-dimethylglycine (*N,N*-DMG) correlated only in controls with good digestive tolerance. Preterm controls with feed intolerance showed a progressive significant decrease of *N*-methylnicotinamide and carnitine. Lactate, betaine, myo-inositol, urea, creatinine, and *N,N*-dimethylglycine discriminated late-onset NEC from controls with good feed tolerance.

**Conclusion:** Our findings are discussed in terms of contributions from nutritional and clinical managements of patients and gut microbiota.

## Introduction

Cellular metabolism plays a crucial role both in health and disease, mirroring interactions between the host genome, environment, and microbiome. Environmental and lifestyle factors as well as traumatic events or diseases, have the potential to alter the individual metabolic phenotype both directly, by inducing perturbations in various metabolic pathways, and indirectly, by promoting epigenetic changes, which in turn lead to changes in gene expression, transcripts, and ultimately in the metabolic profile of a given cell, tissue, or biological fluid (Eicher et al., 2020). However, the organism’s rapid response to any exogenous or endogenous factor altering the cellular and tissue homeostasis (e.g., asphyxia, sepsis, gut dysbiosis) can be unveiled and monitored overt time NEITHER by genomics nor by transcriptomics and proteomics. These “omics” offer specific but tardive information on any biological change; conversely, metabolomics, the science that identifies and quantifies endogenous and exogenous metabolites, represents an “instant omics” capable to provide information on the current status of a living system (Ashrafian et al., 2020). Indeed, changes in the individual metabolic profile occur much earlier than any clinically detectable sign or symptom, and thus, metabolomics is strategic in the context of the precision medicine approach (Karczewski and Snyder, 2018). One of the most important applications of metabolomic studies is the early identification of critically ill newborns at risk of adverse clinical outcomes during their stay in the neonatal intensive care unit (NICU) (Fanos et al., 2018; Bardanzellu et al., 2020; Locci et al., 2020). Necrotizing enterocolitis (NEC) is a life-threatening disease affecting almost exclusively preterm newborns, consisting of abnormal intestinal colonization followed by an immune-inflammatory response leading to the loss of intestinal barrier function and possible perforation of the intestine (Neu and Walker, 2011; Meister et al., 2020; Neu, 2020). The pooled estimated NEC incidence in very low birth weight (VLBW) newborns is approximately 7% (Alsaied et al., 2020), while it is less common in late premature and in full term newborns. NEC’s pathogenesis is primarily marked by an abnormal inflammatory response and necrosis of the gut mucosa along the whole gastrointestinal tract (Niño et al., 2016). Further risk factors include sepsis, enteral formula feeding, prolonged antibiotic exposure, and gut dysbiosis (Raba et al., 2021). Recently, it was demonstrated that NEC is associated with elevated blood levels of CCR9 + CD4 + T cells as well as CCR9 + interleukin-17 (IL-17) producing Treg (previously called regulatory T cells); the histological NEC severity is positively and negatively correlated with their gut and blood concentration, respectively (Ma et al., 2019). As a consequence, the therapeutic modulation of lymphocyte balance may open new perspectives for improving NEC severity and outcome (Nguyen and Sangild., 2019). Recommendations on feeding practices, such as breastfeeding, the proper management of feeding intolerance, the application of feeding guidelines, and the implementation of probiotics with diet, can prevent NEC onset in critically ill newborns admitted in NICU (Bi et al., 2019). Several studies, recently revised in an elegant review (Agakidou et al., 2020), have investigated the metabolic profile of blood, plasma, serum, urine, stools, and intestinal epithelial cells in preterm neonates with NEC, opening new horizons on the molecular mechanisms associated with the disease and searching candidate biomarkers for the early diagnosis and prognosis of NEC. This pilot study aimed to explore the presence of the urinary metabolic signature in VLBW preterm newborns by using a proton nuclear magnetic resonance spectroscopy (^1^H NMR)-based metabolomic approach. We investigated the dynamic changes of the urine metabolome in infants with NEC over the first 2 months of life by collecting samples at different time points, namely before, during, and after the diagnosis. Since all babies with NEC were also affected by feeding intolerance (FI), each of them was matched with two preterm newborns without NEC, the first one with FI and the second one with good digestive tolerance.

## Materials and Methods

### Patients

This case-control study was conducted in the NICU, Hospital de la Croix Rousse (HCR), Hospices Civils de Lyon, Lyon, France. The study was approved by the local Ethics Committee (Comité de Protection des Personnes Sud-Est IV, Lyon) and performed following the approved guidelines. Infant parents signed informed consent forms before participation. We considered eligible for the study VLBW preterm babies recruited prospectively. Eighteen VLBW preterm infants were included: 6 with NEC and feeding intolerance (group 1, NEC); 6 with feeding intolerance without any sign of NEC (group 2, FI); and 6 with good digestive tolerance without NEC (group 3, GDT). We considered feeding intolerance the inability of the baby to ingest and digest enteral nutrition. This condition became clinically evident with the appearance of (a) gastric residues (more than 50% of the ingested food after 2–3 consecutive meals); (b) biliary or hemorrhagic color of residues; (c) abdominal distension with discomfort on palpation and gaseous dilation of the loops of the small intestine (Lucchini et al., 2011). Good feeding tolerance was defined as the ability of the preterm infant to safely ingesting and digesting the prescribed enteral feeding without complications associated with aspiration, infection, and gastrointestinal dysfunction (Shulman et al., 1998). In group 1, three babies developed the disease within the first 3 weeks of life (hereafter called *early-onset* NEC), while the remaining three developed NEC 6–8 weeks after birth (hereafter called *late-onset* NEC). Each baby belonging to group 1 NEC was matched with two babies, the first one belonging to group 2 FI and the second one to control group 3 GDT. In order to reduce uninformative variations that could interfere with the identification of relevant information encoded in the experimental spectral dataset, controls were selected according to matched gestational age (± 1 week), birth weight (± 200 g), and postnatal age at the time of urine sampling (± 7 days). NEC was defined as the presence of clinical evidence fulfilling modified Bell’s stage criteria (Bell et al., 1978; Juhl et al., 2019) and was confirmed by radiological pneumatosis intestinalis. All NEC cases were Bell stage II. Neonates with major congenital abnormalities (including those of the gastrointestinal tract) were excluded as controls from the study. Total parenteral nutrition was used for all infants up to 2–3 weeks of life (Darmaun et al., 2018). As soon as tolerated, enteral feeding was gradually introduced using a milk bank or expressed breast milk provided by their mother. According to the European Milk Bank Association (EMBA) Working Group recommendations (Arslanoglu et al., 2019), milk was fortified in both cases. Fortema® (Bledina, Villefranche-sur-Saône, France) for protein and carbohydrate intakes, Liquigen® (Nutricia, Saint-Ouen, France) for lipid intakes and the multicomponent fortifier Nutriprem® (Bledina, Villefranche-sur-Saône, France) were used. In babies manifesting feeding intolerance, enteral nutrition was interrupted and then gradually reintroduced when clinical conditions went back to normal. During the study period, no change was made in the enteral parenteral nutritional policy.

### Sample Collection and Preparation

Urine samples were collected over approximately two months after birth. In babies with NEC (group 1), urine samples were collected at various intervals: before, during, and after the disease’s onset. In babies belonging to groups 2 FI and 3 GDT, samples were collected at the day of life as close as possible to those collected in babies with NEC. Samples (volume 1–2 ml) were collected using a cotton ball inserted into the disposable diaper; the urine was aspired by a syringe and then transferred to a sterile 2 ml vial and immediately frozen at −80°C until their shipping to the metabolab of the University of Cagliari. Before analysis, 800 μL of thawed urine were transferred into a 1.5 ml centrifuge microtube, and then 8 μL of sodium azide (10% w/w) were added to avoiding any possible bacterial growth. The sample was then centrifuged at 12,000 *g* for 10 min at + 4°C. To stabilize the pH of urine samples, 630 μL of supernatant were mixed with 70 μL of phosphate buffer solution [1.5M KH_2_PO_4_, 1% sodium 3-trimethylsilyl-propionate-2,2,3,3-d4 (TSP, 98 atom% D), pH 7.4]. Finally, 650 μL were placed into a 5 mm wide NMR tube.

### Proton Nuclear Magnetic Resonance Spectroscopy Analysis

The analysis was conducted at 300K by using a Varian UNITY INOVA 500 spectrometer (Agilent Technologies, CA, United States) operating at 499.839 MHz. A standard 1-D pulse sequence NOESY was used with water suppression. For each urine spectrum, a total of 128 scans were collected in 64k data points over a spectral width of 6,000 Hz using a relaxation delay of 2 s, an acquisition time of 1.5 s, and mixing of 0.1 s. Before Fourier transformation, the free induction decay was multiplied with 0.3 Hz exponential line broadening spectra. All spectra were phased, and baseline corrected using MestReNova (Version 8.1, Mestrelab Research SL, Santiago de Compostela, Spain). The chemical shift scale was set by assigning a value of δ = 0.00 ppm to the internal standard TSP signal. After correction for misalignments in chemical shift, primarily due to pH-dependent signals and deleting the regions containing the water and TSP signals, the NMR spectra were binned into 0.0025 ppm buckets over a chemical shift range of 0.5–9.5 ppm. Bins were normalized to the sum of total spectral area to compensate for the overall concentration differences and used as a dataset (97 x 4,423) for multivariate analysis. The assignment of the metabolites in the ^1^H NMR spectra was performed according to literature data (Diaz et al., 2016; Scalabre et al., 2017), the Human Metabolome database, available at http://www.hmdb.ca (Wishart et al., 2018), and Chenomx NMR suite 8.1 software (evaluation version, Chenomx, Edmonton, Canada). All the samples were analyzed simultaneously.

### Data Processing and Statistical Analysis

For multivariate statistical analysis, data were Pareto scaled (Misra, 2020). Multivariate statistical analysis of the NMR dataset consisted of principal component analysis (PCA), orthogonal projection to latent structures (OPLS) regression, and orthogonal projection to latent structures discriminant analysis (OPLS-DA) supported by the SIMCA software (version 16.0, Umetrics, Umeå, Sweden). OPLS was used in the case of a continuous Y-matrix (i.e., multiple time points) and OPLS-DA for identifying discriminant metabolites in a pairwise comparison between case and control groups. The quality of OPLS and OPLS-DA models were evaluated through the following parameters: the cumulative values of total Y explained variance, i.e., goodness of fit (R^2^Y), and the Y predictable variation, i.e., the goodness of predictability (Q^2^). The latter was extracted by the default method of 7-fold internal cross-validation of SIMCA. Additionally, the models were tested for overfitting using permutation testing (*n* = 400). The models’ significance was further assessed by an ANOVA based on the cross-validated predictive residuals (CV-ANOVA) with a *p*-value ≤ 0.05 (Eriksson et al., 2008). The models were considered valid if the permutation test and the CV-ANOVA test were significant. The variables with the most significant contributions to OPLS and OPLS-DA models were identified by exploring the correlation coefficients line plots by following two criteria: absolute *p* and *p(corr)* values were set to be greater than 0.05 and 0.5, respectively. *p*-value represents each variable’s importance and *p(corr)* its reliability (Cloarec et al., 2005). The univariate statistical analysis was performed by the GraphPad Prism Statistics software package, version 8.1.2 (GraphPad Prism Software Inc., San Diego, CA, United States), to measure the Pearson’s correlation coefficient between metabolites and postnatal time and compare the variation in the abundance of discriminant metabolites between groups. The magnitude of variation was evaluated by calculating the effect size (ES) adjusted for small sample number (Berben et al., 2012). Effect sizes were classified small between 0.2 and 0.5, medium between 0.5 and 0.8, and large when greater than 0.8. The non-parametric Mann-Whitney U test was used for the univariate statistical approach; a *p-*value ≤ 0.05 was considered statistically significant.

## Results

**FIGURE 1:**
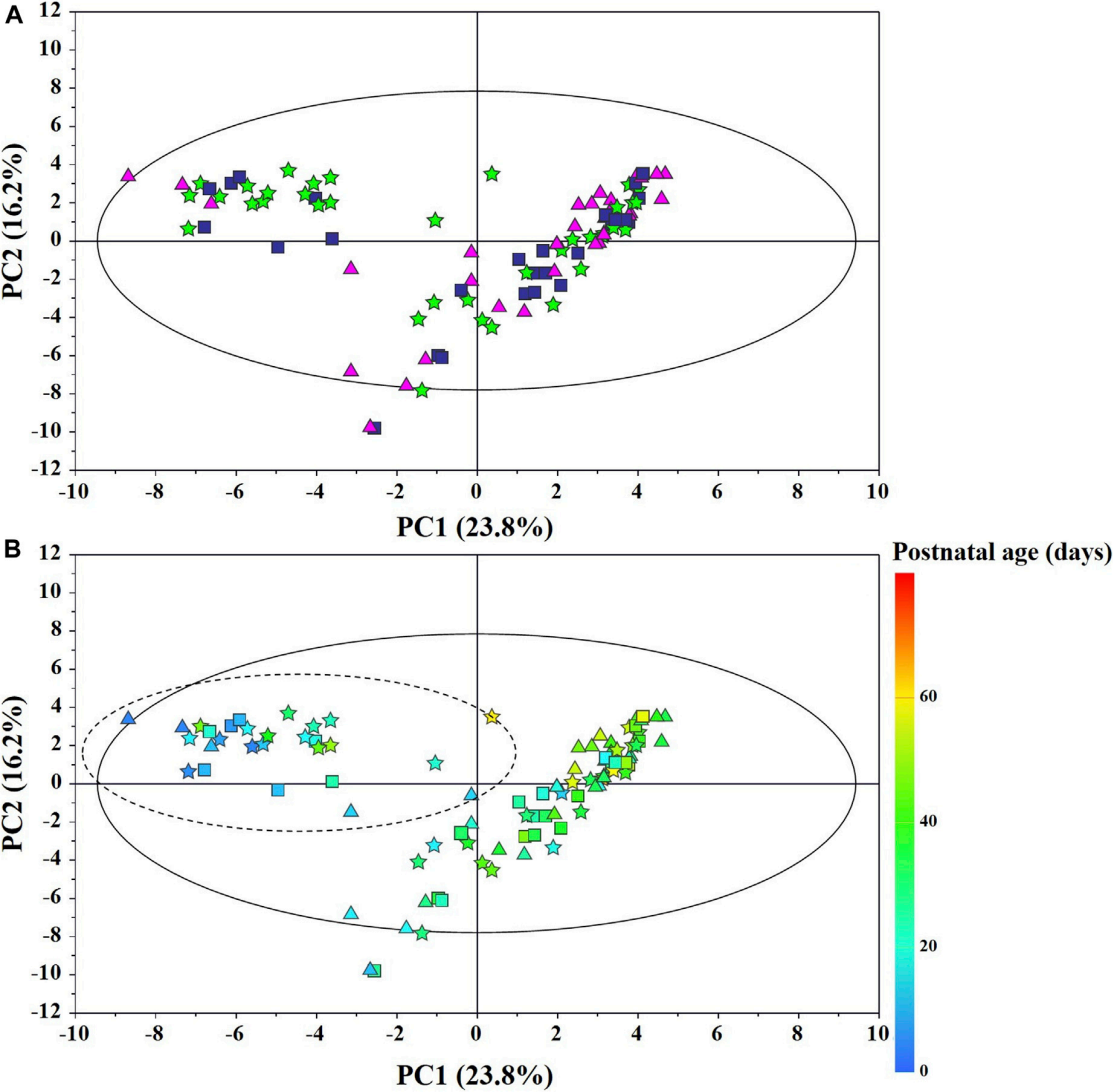
PCA scores plot from the model built with the ^1^H-NMR spectra of infant urine samples: **(A)** ★, group 1 NEC; ■, group 2 FI (feed intolerance without NEC); ▲, control group 3 GDT (good digestive tolerance without NEC); **(B)** Scores are coloured according to the postnatal age. Samples in the dotted circle were collected during total parenteral nutrition (TPN).

**FIGURE 2:**
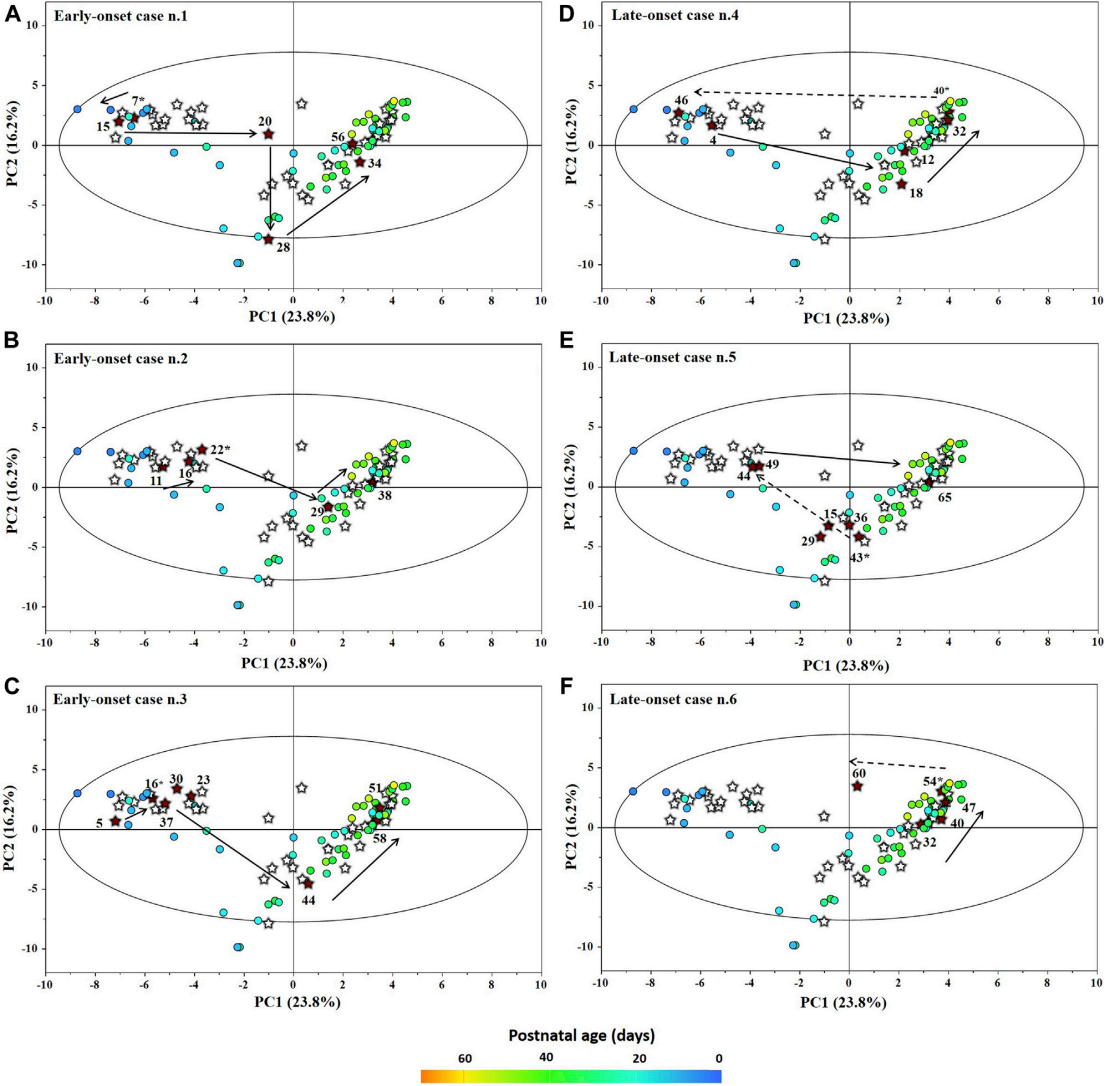
Temporal trajectories (obtained from the model built with the ^1^H-NMR spectra) of each individual early-onset **(A–C)** and late-onset **(D–F)** NEC baby in the PC1 vs PC2 scores plot. FI and GDT (o); NEC (★). Scores are colored according to the postnatal age. Numbers denotes the post-natal age of NEC at the time of sampling and the asterisk marks the day of the disease onset.

**FIGURE 3:**
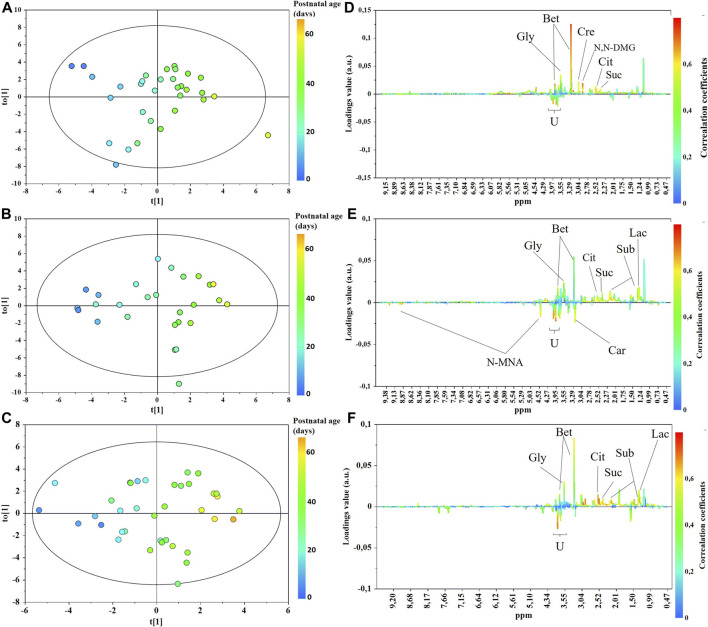
OPLS scores **(A–C)** and loadings line **(D–F)** plots of the ^1^H-NMR urine spectra from control group 3 GDT **(top)**, group 2 FI **(middle)**, and group 1 NEC **(bottom)**. The scores are coloured according to the postnatal age. Abbreviations: Bet, betaine; Car, carnitine; Cre, creatinine; Cit, citrate; *N,N*-DMG, *N,N*-dimethylglycine; *N*-MNA, *N*-methylnicotinamide; Gly, glycine; Lac, lactate; Sub, suberate; Suc, succinate.

**FIGURE 4:**
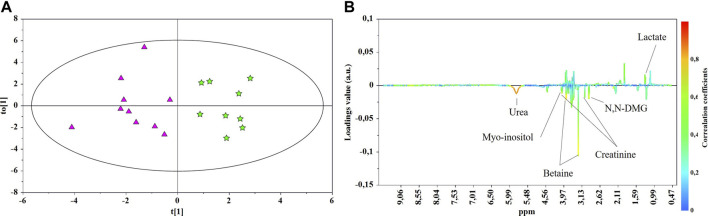
OPLS-DA scores **(A)** and correlation loading **(B)** plots for the pair-wise comparison between the *late-onset* NEC subgroup (★) and control group 3 GDT (▲). ^1^H NMR spectra of urine samples collected just prior to and at the days of NEC diagnosis were analyzed.

**FIGURE 5:**
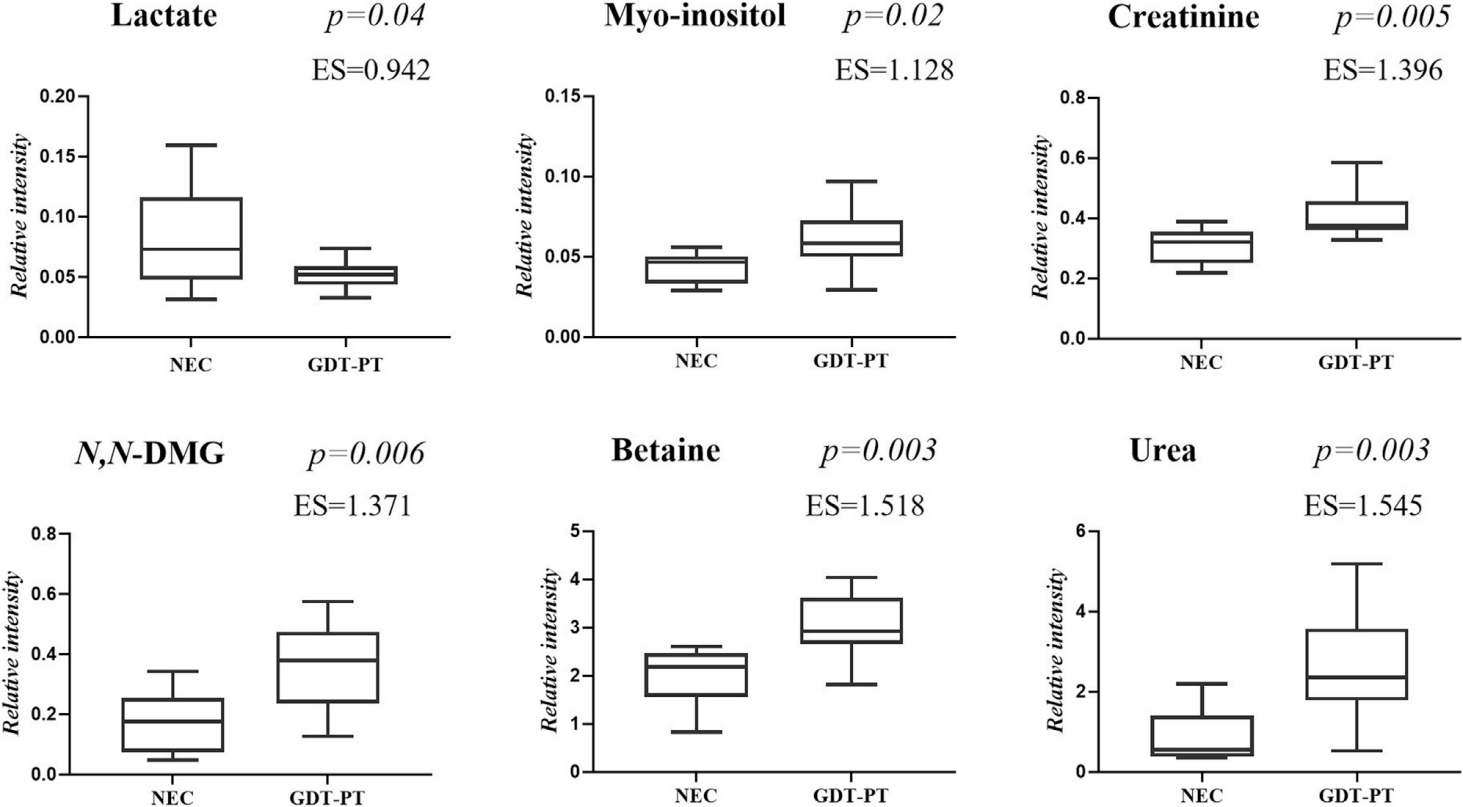
Selected metabolites discriminating control group 3 GDT and *late-onset* NEC subgroup the day before the onset of the disease and at the disease onset. Values in the box plots are shown as the normalized peak areas of the metabolites.

The main characteristics of the study population are reported in Table 1. Group 1 NEC does not significantly differ from group 2 FI and 3 GDT for gestational age, birth weight, mean Apgar scores at 5 min (*p* > 0.05). The male:female ratio was 1:1 for each group. A total of 97 urine samples were collected during the first 2 months of life: 38 in group 1 NEC (18 *early-onset* NEC, 20 *late-onset* NEC), 27 in group 2 FI, and 32 in control group 3 GDT. A preliminary PCA analysis was performed for searching any inherent separation among samples and the presence of outliers. The total amount of variance explained by the first two principal components (PCs) was 40%. The PC1 *vs.* PC2 scores plot clearly indicates the absence of any cluster (Figure 1A). Conversely, the scores plot unveils a similar, unidirectional temporal trend for groups 2 FI and 3 GDT, scores shifting from the left to the right side of the PC1 axis as age increases (Figure 1B). The temporal shift of the urine metabolome of the *early-onset* NEC subgroup follows the same trajectory along the PC1 axis as those exhibited by groups 2 and 3 (Figures 2A–C). On the other hand, the time course of the *late-onset* NEC subgroup is similar to that of the other groups, but only until the diagnosis of the disease; soon after, the trajectory inverts the direction, approaching the metabolic profile of the first days of life (Figures 2D–F). The PCA loadings plot revealed that this behavior was mainly related to the urinary gluconate concentration (Supplementary Figure S1). Indeed, in the urine sample of the *late-onset* NEC subgroup, rigorously collected after the diagnosis, the content of gluconate was much higher than that found in the urine of the same babies collected just before the diagnosis; moreover, gluconate concentration was comparable with that observed in the urine collected during the first day of life in all the neonates. Gluconate is a nutrient degraded by gluconokinase to generate 6-phosphogluconate, playing a crucial physiological role (Ramachandran et al., 2006; Riganti et al., 2012); however, during the total parenteral nutrition (TPN) at birth and after the clinical diagnosis of NEC, the intravenous calcium administration was a source of exogenous gluconate in babies with NEC. The subgroup of *early-onset* NEC babies developed the disease during TPN, while in the *late-onset* subgroup, NEC was diagnosed during enteral nutrition (EN). In detail, the latter received two cycles of TPN, namely at birth and after the onset of NEC, alternated by an EN cycle. Based on these findings, the analysis of the temporal trajectories of *late-onset* NEC scores in the PCA model showed that the inversion of the trend observed after the diagnosis was associated with the introduction of the second cycle of TPN therapy, leading to the reasonable conclusion that the nutritional intervention may be the main source of this dynamic modification. Since the urine NMR spectra of all infants under TPN showed very intense signals due to exogenous gluconate, we removed these signals before statistical analysis to avoid any TPN contribution to the spectral profile. A more detailed insight into the time dependence of urine metabolome of group 1 NEC and groups 2 and 3 was undertaken by OPLS regression by using the ^1^H NMR urine spectral data as an independent variable and the postnatal sampling days as Y-variable. An OPLS model was built separately for each group. Model performances are summarised in Table 2, while Figure 3 depicts the corresponding scores and loading plots. The models built for group 2 FI and 3 GDT demonstrated good modeling and predictive abilities, while a lower but acceptable predictivity characterized the model for group 1 NEC. The robustness of the models was validated by the permutation test (*n* = 400) and CV-ANOVA. The corresponding loadings plots (Figures 3D–F) allow identifying the most significant metabolic signature associated with postnatal age. Metabolites positively correlating with postnatal age were: betaine, glycine, citrate, and succinate in all the groups; *N,N*-dimethylglycine (DMG) in control group 3 GDT; suberate and lactate in group 1 NEC and group 2 FI; creatinine in control group 3 GDT only. Besides, unassigned resonances at δ = 3.95 and δ = 3.74 were inversely correlated with postnatal age in all the groups, while carnitine and *N*-methylnicotinammide (*N*-MNA) only in group 2 FI. These time-dependent changes were also investigated by the univariate statistical analysis; in particular, the Pearson’s correlation coefficient (r) was computed. Results confirmed the statistical significance of the findings mentioned above- (*p* < 0.05) (Supplementary Table S1). To identify the metabolic signature(s) associated with NEC, the two subgroups of *early-*and *late-onset* NEC were compared with the corresponding matched groups 2 and 3 by using the OPLS-DA approach. We included only spectra of urine collected either immediately before or at the diagnosis time, based on the assumption that they may provide more information on the presence of metabolic perturbations associated with the disease. In Table 3, we reported the quality parameters of the pairwise OPLS-DA models. No model comparing the *early-onset* NEC subgroup with groups 2 and 3 was found significant; conversely, the model comparing the *late-onset* NEC subgroup with control group 3 GDT showed a significant group separation (*p* = 0.02) as reported in Figure 4A. The analysis of the OPLS-DA loadings plot (Figure 4B) showed the leading metabolites responsible for sample discrimination, providing an assessment of the main statistically significant differences between the two groups: lactate was more abundant in the *late-onset* NEC subgroup, while *N,N*-DMG, betaine, creatinine, myo-inositol, and urea were more abundant in group 3. These findings were further supported by the univariate statistical analysis for assessing significant differences in the relative content of these metabolites between the two groups (Figure 5).

**TABLE 1:** Characteristics of study population.

Variable, description	NEC (*n* = 6)	Controls (*n* = 12)	
		FI-PT (*n* = 6)	GDT-PT (*n* = 6)
Gestational age (weeks, mean ± SD)	27.1 ± 1.6	27.2 ± 1.3	27.7 ± 1.6
Male/Female, n	3/3	3/3	3/3
Birth weight (g, means ± SD)	1,016 ± 104	920 ± 104	950 ± 65
Cesarean section delivery, n	2	5	3
IUGR, n	1	4	1
Apgar score: ≤5 at 5 min, n	1	2	2
Early-onset of NEC (< 25 days), n	3	/	/
Late-onset of NEC (> 40 days), n	3	/	/
Antibiotics, n	6	6	6

Abbreviation: FI-PT, preterm with feed intolerance; GDT-PT, preterm with good digestive tolerance; IUGR, intrauterine growth restriction; NEC, necrotizing enterocolitis; SD, standard deviation.

**TABLE 2:** Statistical parameters of the OPLS models derived from the ^1^H-NMR spectra of urine samples from group 1 NEC, group 2 FI, and group 3 GDT cases.^a^

Data set	R^2^Y	Q^2^Y	Permutation test^b^		*p*-value^c^
			R^2^ *Y* intercept	Q^2^ *Y* intercept	
Group 1 NEC	0.743	0.398	0.509	−0.463	2.01 ^**.**^ 10^−3^
Group 2 FI	0.813	0.567	0.531	−0.545	7.26 ^**.**^ 10^−3^
Group 3 GDT	0.880	0.638	0.461	−0.430	1.05 ^**.**^ 10^−5^

a Gluconate signals were removed from the dataset.

b n = 400.

c *p-value* obtained from cross validation ANOVA (CV-ANOVA). NEC, necrotizing enterocolitis; FI, food intolerance; GDT, good digestive tolerance.

**TABLE 3:** Statistical parameters for the OPLS-DA models built for the pairwise comparison between cases and controls.^a^

Pairwise comparison				Permutation test		
	R^2^X	R^2^Y	Q^2^Y	R^2^ *Y* intercept	Q^2^ *Y* intercept	*p*-value
Early-onset NEC *vs* FI	0.325	0.906	0.263	0.860	0.114	1
Early-onset NEC *vs* GDT	0.395	0.811	0.023	0.740	−0.034	0.99
Late-onset NEC *vs* FI	0.434	0.844	0.461	0.624	−0.554	0.07
Late-onset NEC *vs* GDT	0.273	0.805	0.624	0.627	−0.606	0.02

a The models were considered valid only if the permutation test and *p-value* obtained from the cross validation ANOVA (CV-ANOVA) test (*p* < 0.05) were satisfied at the same time. NEC, necrotizing enterocolitis; FI, food intolerance; GDT, good digestive tolerance.

## Discussion

Despite the impressive body size of literature on clinical metabolomics-based studies in human disease, the number of metabolomics-based studies on NEC is small, and the identification of reliable candidate omics-based biomarkers for the prediction and the early diagnosis of NEC is still far from being definitive. After the exclusion of metabolomics-based studies enrolling babies with sepsis (both early-onset and late-onset sepsis), approximately ten studies have utilized metabolomics alone or combined with other omics in preterm newborns with NEC; five were based only on metabolomics in various biological fluids, including serum (Wilcock et al., 2016; Wang et al., 2019), stools (Rusconi et al., 2018), urine (Thomaidou et al., 2019), and dry blood spots (Sinclair et al., 2020). A study combined metabolomics with proteomics in serum samples (Stewart et al., 2016a), and four studies integrated metagenomics with metabolomics in urine (Morrow et al., 2013) and stools (Stewart et al., 2016b; Wandro et al., 2018; Brehin et al., 2020). The heterogeneity of patient cohorts, patients and samples size, samples type, analytical methods, length of patient monitoring, diagnostic criteria of NEC, nutrition, and the presence of potentially confounding factors such as comorbidities (sepsis, bronchopulmonary dysplasia) hampers an adequate comparison between our results and those previously published. Overall, the time-dependent shift of scores observed in all the babies enrolled in our study confirms the dynamic postnatal metabolic change due to the influence of age, height, and weight progression, with the concomitant development and maturation of organs and tissues, as previously found elsewhere (Cesare Marincola et al., 2016; Scalabre et al., 2017).

Betaine, a trimethylated glycine derived either from diet or the oxidation of choline, increased over time in all the three groups of infants, while *N,N*-dimethylglycine (*N,N*-DMG), derived from the loss of a methyl group from betaine, increased only in control group 3, GDT. Betaine and *N,N*-DMG are methyl donors in several metabolic pathways, including the homocysteine and the DNA methylation. Betaine is involved in osmotic homeostasis, protecting cells from dehydration and kidneys from injuries; betaine is also an anti-oxidant and is involved in neurodevelopment and immune functions. In the urine of healthy adults, betaine is almost completely absent; conversely, in healthy newborns’ urine, high betaine levels are usually present, reflecting dietary choline disposal (Davies et al., 1988). Breast milk is an important dietary source of choline which is essential during infant growth and development. Interestingly, urine betaine and *N,N*-DMG levels were significantly reduced in the subgroup of babies with *late-onset* NEC (20 samples) compared with control group 3 GDT (32 samples), confirming previous findings that associated the reduction of urine betaine with NEC (Thomaidou et al., 2019). Data from the literature evidence that urine betaine was found decreased over the first 48-h of life in various groups of full-term infants with impaired growth and this trend was followed by the increase of betaine at the end of the first week of life (Marincola et al., 2015). Conversely, high urine levels of betaine were observed in full-term infants with congenital cytomegalovirus infection (Fanos et al., 2013) and hypoxic-ischemic encephalopathy (Locci et al., 2018). Thus, the decrease of urine betaine in babies with *late-onset* NEC may be associated with prematurity and kidney dysfunction rather than with sepsis and inflammation. In our preterms, the influence of infant growth and maturation over time is reflected by the positive correlation between postnatal age and glycine, succinate, and citrate. However, in the group of babies with *early-* and *late-onset* NEC (38 samples), urinary succinate and citrate were significantly reduced (fold change −0.161 and −0.163, respectively) compared to control group 3 GDT. On the other hand, they were closely comparable between group 2 FI (27 samples) and group 3 GDT (32 samples). Conversely, urine glycine abundance was almost equal between group 1 NEC and control group 3 GDT and significantly increased in group 2 FI (fold change 0.161). The reduction in succinate and citrate in group 1 NEC may be related to the impairment of the tricarboxylic acid (TCA or Krebs) cycle in babies with NEC, leading to decreased carbohydrates, amino acids, and lipids availability. Indeed, as newborns gain weight during the early postnatal age, the increase of urine succinate and citrate may reflect the high metabolic turnover due to the increasing energy demand (Moltu et al., 2014; Scalabre et al., 2017).

A positive correlation between urine creatinine and postnatal age was observed in control group 3 GDT but not in group 1 NEC; a weak correlation was also found in group 2 FI. Creatinine abundance was significantly lower in group 1 NEC compared with group 3 GDT (fold change −0.74) and with group 2 FI (fold change −0.68), confirming similar results (fold change −0.35) previously published elsewhere (Thomaidou et al., 2019). Based on our results and data from the literature, we could argue that in preterm babies with good digestive tolerance (group 3 GDT), the positive correlation between creatinine and postnatal age is due at least to two factors: the progressive maturation of the kidney leading to the increase in glomerular filtration rate (GFR), even though slower than in full-term infants (Gubhaju et al., 2014), and the progressive increase of muscle mass. The latter is closely related to the rate of protein synthesis, which depends on feeding (Davis and Fiorotto, 2009). In groups 1 NEC and 2 FI, the replicated interruptions overtime of the enteral feeding, in response to clinical symptoms of food digestive intolerance, slows down the synthesis of proteins, with a negative consequence on the growth of organs and tissues, such as the kidney and muscle mass, and ultimately with the decrease of urine creatinine excretion. In the urine samples of the subgroup of babies with *late-onset* NEC (20 samples), we observed high lactate levels compared with those of the control group 3 GDT, especially close to the onset of the disease. A possible explanation may be the impaired TCA production of energy associated with NEC. In babies with *early-onset* NEC and in those with food intolerance (group 2 FI), the predominance of hyperlactatemia over the first 48–72 h of life in preterm infants may reduce differences between groups (Junior et al., 2021); later, lactate levels may better discriminate critically ill preterm infants with NEC or other acute diseases, from preterm infants with non-severe acute disease. Increased levels of lactate may also originate from different sources. Lactate produced by human metabolism is primarily the levorotatory isomer l-lactate; conversely, d-lactate is prevalently produced by bacterial fermentation of undigested carbohydrates in the gastrointestinal tract, and only a small fraction of this isomer is endogenously formed from methylglyoxal through the glyoxalase system (Adeva-Andany et al., 2014). High levels of d-lactate have been found in the urine and plasma of preterm babies with NEC (Garcia et al., 1984; Lei et al., 2016); this finding suggests that d-lactate may be considered an index of increased enteric bacterial activity (Grishin et al., 2013). Although ^1^H NMR is unable to distinguish the lactate enantiomers, we cannot rule out that the high abundance of lactate in the urine of infants with *late-onset* NEC may derive at least in part from the accumulation of d-lactate. The positive correlation between lactate and postnatal age in group 2 FI and in the subgroup *late-onset* NEC, together with the concomitant decrease in *N*-methylnicotinamide, seem to confirm an imbalance of the host−microbial metabolism in these infants. Indeed, *N*-methylnicotinamide has been utilized as an index of the suppression of the gut microbiome in an experimental study in an animal model on NEC (Jiang et al., 2017).

Myo-inositol, an inositol stereoisomer mediating cell signal transduction in response to a variety of hormones, neurotransmitters, and growth factors, was decreased in the urine of babies with *late-onset* (fold change −0.20), but not *early-onset* NEC, and in the urine of babies with FI (group 2, fold change −0.17), compared with control group 3 GDT. Factors such as kidney impairment and perturbances in the metabolism of glucose and lung surfactant influence the urinary level of myo-inositol in preterm infants; however, myo-inositol is a natural constituent of breast milk and is commonly added to formula milk (Brion et al., 2021). Therefore, the nutritional intake strongly influences the myo-inositol concentration. It is reasonable to argue that the dietary restriction, applied after multiple episodes of feeding intolerance prior to the onset of the NEC and their management during the disease, contributes to decreasing urine myo-inositol in *late-onset* NEC and FI groups. Our result confirms similar results previously reported elsewhere (Thomaidou et al., 2019); it is also supported by the simultaneous decrease of urea, a nutritional index reflecting the protein intake, in babies with NEC and FI.

Our study is affected by several limitations. First, the small number of preterm infants limits the strength of results; however, this is a pilot study. Second, the lack of any taxonomic characterization of the gut microbiota hampers to elucidate the significance of metabolic alterations originating from dysbiosis and abnormal gut microbiota fermentation. Third, this single-center study hampers the recruitment of a large number of patients and the comparison of the effects of the therapeutic management on the urine metabolome between different centers. Fourth, this study adopted a single analytical platform. Combining the highly quantitative and reproducible nature of ^1^H NMR spectroscopy with the high sensitivity and specificity of MS may improve the panels of detectable metabolites, and potentially the reliability and accuracy of statistical models. A further limitation is the lack of ANOVA for repeated measurements (RM ANOVA); however, this limitation does not hamper the identification of candidate biomarkers for NEC, derivable by the OPLS-DA model. The strengths of this study are the analysis of longitudinal data and the classification of infants with NEC in *early-onset* (sample size = 18) and *late-onset* NEC (sample size = 20). In a previous study, babies with NEC were divided based on the gut microbiota composition (Morrow et al., 2013); unfortunately, a specific bacterial fingerprint associated with NEC was never identified unambiguously. Thus, even that study reported no definitive metabolic data. Overall, previous metabolomics-based studies on preterm infants with NEC are often inconclusive, even when metabolomics was combined with proteomics or microbiomics; (Stewart et al., 2016a; Wilcock et al., 2016; Brehin et al., 2020). Our study confirms that the urine metabolome of infants with NEC is significantly different from that of preterms infants with food intolerance but without NEC and from that of preterm infants with good digestive tolerance. However, the identification of robust candidate biomarkers of NEC requires the system biology approach based, at least, on metabolomics and microbiomics for defining an early accurate diagnosis of the disease and predicting the risk of an adverse clinical outcome much earlier than the clinical onset of the disease.

## Data Availability

The raw data supporting the conclusion of this article will be made available by the authors, without undue reservation.
