# Supplementary material for: Urine NMR Metabolomics Profile of Preterm Infants With Necrotizing Enterocolitis Over the First Two Months of Life: A Pilot Longitudinal Case-Control Study
Source: Front Mol Biosci. 2021 Jun 15;8:680159. doi: 10.3389/fmolb.2021.680159 (PMC8239193; doi:10.3389/fmolb.2021.680159)
Supplement: Supplementary file 1 [file Table1.pdf]

Supplementary Table 1. Pearson's correlation coefficients determined for postnatal time and metabolites selected from the OPLS analysis.

	**Correlation coefficients**		
**Metabolites**	**GDT**	**NEC**	**FI**
betaine	0.768****	0.477**	0.244**
carnitine	-	-	-0.468*
citrate	0.675****	0.756****	0.706****
creatinine	0.542**	-	-
N,N-DMG	0.771****	-	-
glycine	0.259*	0.431*	0.429*
lactate	-	0.430*	0.675***
N-MNA	-	-	-0.604***
suberate	-	0.659****	0.736****
succinate	0.609***	0.501**	0.607***

* $p<0.05$; ** $p<0.01$;***<0.001; **** $p<0.0001$
