# Supplementary material for: Urine NMR Metabolomics Profile of Preterm Infants With Necrotizing Enterocolitis Over the First Two Months of Life: A Pilot Longitudinal Case-Control Study
Source: Front Mol Biosci. 2021 Jun 15;8:680159. doi: 10.3389/fmolb.2021.680159 (PMC8239193; doi:10.3389/fmolb.2021.680159)
Supplement: Supplementary file 2 [file Image1.pdf]

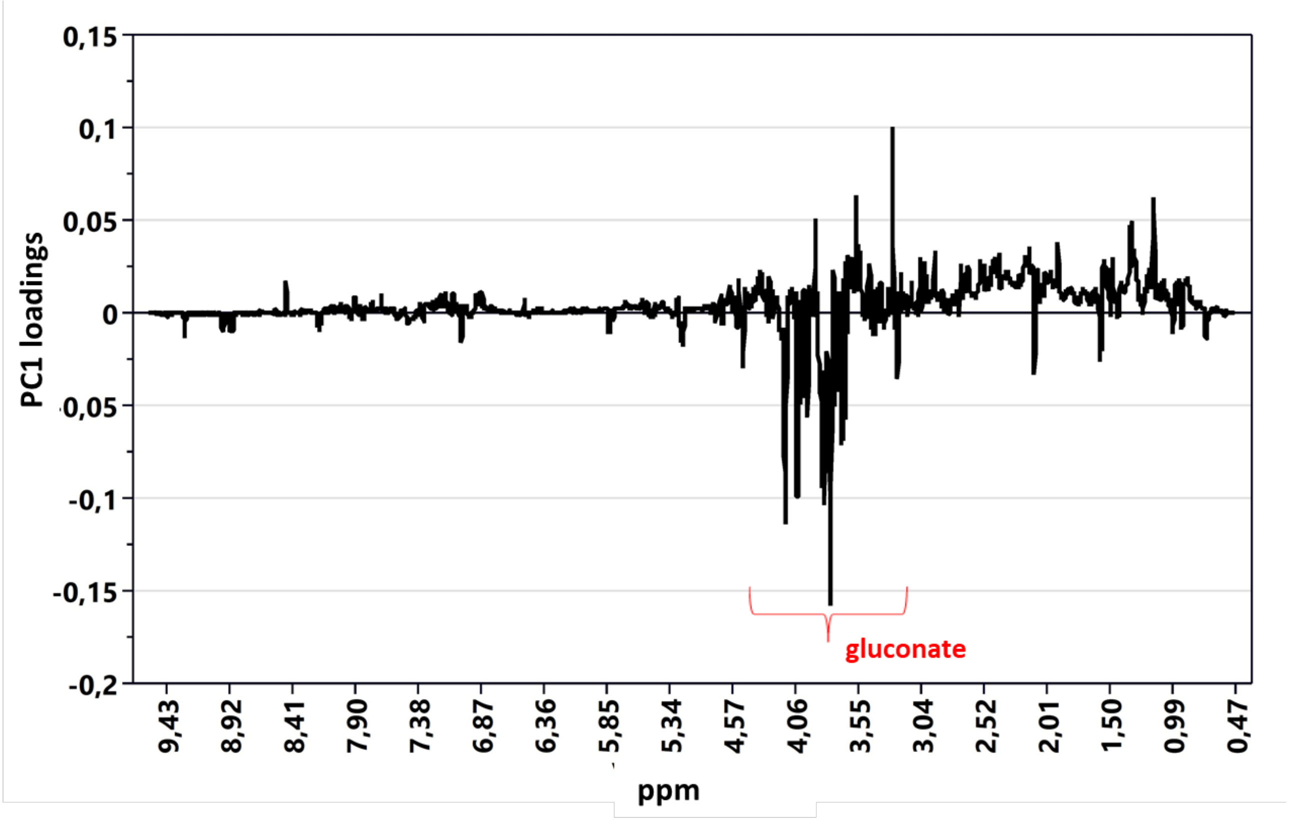

Supplementary Figure 1. PC1 loadings plot for the PCA model built with all the ^{1}H-NMR 45 spectra of infant urine samples.
